# A potential therapeutic drug for osteoporosis: prospect for osteogenic LncRNAs

**DOI:** 10.3389/fendo.2023.1219433

**Published:** 2023-08-03

**Authors:** Fanjin Meng, Yang Yu, Ye Tian, Meng Deng, Kaiyuan Zheng, Xiaolan Guo, Beilei Zeng, Jingjia Li, Airong Qian, Chong Yin

**Affiliations:** ^1^ Department of Clinical Laboratory, Department of Oncology, Department of Rehabilitation Medicine, Ministry of Science and Technology, Affiliated Hospital of North Sichuan Medical College, Nanchong, China; ^2^ Department of Laboratory Medicine, Translational Medicine Research Center, North Sichuan Medical College, Nanchong, China; ^3^ School of Pharmacy, Tianjin Medical University, Tianjin, China; ^4^ Lab for Bone Metabolism, Xi’an Key Laboratory of Special Medicine and Health Engineering, Key Lab for Space Biosciences and Biotechnology, Research Center for Special Medicine and Health Systems Engineering, NPU-UAB Joint Laboratory for Bone Metabolism, School of Life Sciences, Northwestern Polytechnical University, Xi’an, Shaanxi, China

**Keywords:** osteogenic, lncRNA, homology, miRNA, bone formation

## Abstract

Long non-coding RNAs (LncRNAs) play essential roles in multiple physiological processes including bone formation. Investigators have revealed that LncRNAs regulated bone formation through various signaling pathways and micro RNAs (miRNAs). However, several problems exist in current research studies on osteogenic LncRNAs, including sophisticated techniques, high cost for *in vivo* experiment, as well as low homology of LncRNAs between animal model and human, which hindered translational medicine research. Moreover, compared with gene editing, LncRNAs would only lead to inhibition of target genes rather than completely knocking them out. As the studies on osteogenic LncRNA gradually proceed, some of these problems have turned osteogenic LncRNA research studies into slump. This review described some new techniques and innovative ideas to address these problems. Although investigations on osteogenic LncRNAs still have obtacles to overcome, LncRNA will work as a promising therapeutic drug for osteoporosis in the near future.

## 1 Introduction

Osteoporosis, an emerging treat of human health, is a systemic bone disease that can be caused by complex factors (1, 2). Growing evidence has shown that the reduction in osteogenic differentiation of bone mesenchymal stem cells (BMSCs) would lead to decreased bone formation, ultimately resulting in osteoporosis. BMSC osteogenic differentiation is a complex physiological process regulated by multiple factors, among which long non-coding RNAs (LncRNAs) have been proved to play essential roles. LncRNA is a category of non-coding RNA with more than 200 nucleotides (3). LncRNAs have been shown to play important roles in regulating various physiological and pathological processes including embryonic development, organ growth, and diseases.

LncRNAs are also indispensable regulators for osteogenesis. Multiple LncRNAs, including LINC01119 (4), SNHG5 (5), KCNQ1OT1 (6), H19 (7), ODSM (8, 9), Crnde (10), MALAT1 (11), and PMIF (12), have been reported to have key regulatory roles in osteogenesis. These investigations provided important references for the mechanisms of osteoblast differentiation and bone formation and were also strong support for LncRNA as a therapeutic drug or target for osteoporosis. However, as research studies on LncRNA continue, several problems emerge, such as techniques for *in vivo* research and low homology of LncRNA among different species. These constituted significant obstacles for LncRNA to truly become a nucleic acid therapy drug. Numerous researchers are trying to solve these problems, and we have also proposed our ideas, which we will briefly describe in this article.

## 2 Osteoporosis and its treatment

Osteoporosis is a long-term disease characterized by deterioration of bone microstructure, loss of bone mass, and increase in bone fragility, which further lead to pain, spinal deformation, and fragility fracture (1, 2). Multiple factors are involved in the pathogenesis of osteoporosis, including heredity, nutrition, aging, and postmenopausal hormone disorder. Essentially, osteoporosis is a disease caused by decreased osteogenesis, which is the outcome of the joint action of osteoblasts and osteoclast. The commonly used medicines for osteoporosis often function in reducing the activity of osteoclast, such as alendronate sodium, etidonate disodium, and clodronate disodium. However, the decreased osteogenesis was more closely correlated with the reduction in the osteogenic differentiation of BMSCs, which could be regulated by multiple signaling pathways and factors including LncRNA. Therefore, investigations on the role of LncRNA in the regulation of BMSC differentiation and osteogenesis are of great significance in gaining insights on the pathogenesis and treatment of osteoporosis.

### 2.1 Current osteogenic LncRNA research studies

Many researchers have reported the role of LncRNA in regulating osteogenic cell differentiation and bone formation. For example, Wu et al. demonstrated that LncRNA AC132217.4 can promote osteogenic differentiation of mesenchymal stem cell through IGF-AKT signaling pathway (13); Gao et al. found that LINC01119 negatively regulated osteogenic differentiation of MSC through targeting FZD4 by Wnt pathway (4); Han et al. found that LncRNA SNHG5 can promote BMSC osteogenic differentiation through miR-212-3p/GDF5/SMAD pathway (5); Yuan et al. reported that LncRNA PGC1β-OT1 promoted osteogenic differentiation of MSCs (14). Wang et al. found that lncRNA KCNQ1OT1 promoted bone formation by inhibiting miR-98-5p/Tbx5 axis (6). Ren et al. found that LncRNA LIOCE promoted bone formation via enhancing Osterix (15). Behera et al. found that exosome-derived LncRNA-H19 bond with miR-106, thereby activating Angpt/Tie2-NO pathway to enhance mesenchymal cell osteogenic differentiation and angiogenesis to promote bone formation (16). Li et al. indicated that LncRNA PMIF hindered BMSC to migrate to the osteogenic region and inhibited bone formation, whereas targeted delivery of PMIF small interfering RNA (siRNA) can rescue bone formation in aging osteoporosis mouse model (12).

Moreover, our previous studies had reported several LncRNAs that function in bone formation inhibition, including AK016739, AK039312, AK079370, and Lnc-DIF (17–19). The abovementioned studies all elaborated the critical roles of LncRNAs in regulating bone formation and provided essential basis on osteogenic research studies (7–10, 20–23). However, some problems exist in current osteogenic LncRNA research studies.

## 3 The existing problems in research studies on osteogenic LncRNA

### 3.1 Length of LncRNAs

The length of LncRNAs poses significant challenges for investigation. LncRNAs that are longer than 3,500 nucleotides (nt) might be difficult to be inserted into vectors and transfected into cells. Whereas, for LncRNAs that are shorter than 800 nt, siRNA designing would be hard, which made it unable to determine its function. Thus, in most research studies, only LncRNAs with length between 800 and 3,500 nt were selected.

### 3.2 Difficulties in *in vivo* research studies

At present, *in vivo* experiments for osteogenic LncRNA are still relatively difficult. The main obstacle is the precise delivery of siRNAs or LncRNAs to the bone formation surface, which is required for the regulation of LncRNA expression in bone tissue. This means complicated experimental skills and massive cost of nucleic acid and vector.

### 3.3 Low homology of LncRNA

Owing to the low homology of LncRNAs among different species, functional studies of LncRNAs still have some limitations. Most LncRNAs derived from model animals are hard to be directly used for therapy of osteoporosis and other bone diseases. Although human-derived LncRNA research studies mostly confined to cellular experiments due to the particularity of skeletal diseases, it is not possible to use *in vivo* animal models to further verify its bone formation function. Therefore, many current research studies on osteogenesis-related LncRNAs have some way to go before providing therapeutic medicine for osteoporosis.

## 4 Methods of resolution

### 4.1 LncRNA *in vivo* research

Compared with *in vitro* research, the *in vivo* research for LncRNA in osteogenesis is much more difficult. For *in vitro* research of bone formation, one can manipulate LncRNA expression level in cultured osteogenic cells simply by transfecting siRNAs or vectors. However, to change the LncRNA expression level in bone tissue, transfection reagents need to have the ability to deliver siRNAs or vectors to bone formation surface and to maintain at least four hours. Furthermore, *in vivo* experiments require large amount of transfection reagents and nucleic acid, which means high cost.

To solve this problem, in our previous studies, si-AK016739 and si-AK045490 were subcutaneously injected to mouse calvaria (19, 24). This technique enabled nucleic acid to stay for sufficient time on mouse calvarial osteogenic surface. Furthermore, the injection is just localized to a small area of mouse calvaria, therefore greatly reducing the amount of transfection reagents and nucleic acid. One month after treatment, mice were labeled with Calcein before sacrifice. Then, calvaria was collected, embedded in opti-mum cutting temperature compound (OCT), and dissected with 4 μm in thickness. Mineral apposition rate was measured using fluorescence microscopy (19). This method could clearly display alterations in mouse bone formation after the LncRNA expression was manipulated. Moreover, it greatly reduced reagent and time cost with more simplified techniques.

However, aging osteoporosis and postmenopausal osteoporosis mostly occur at weight-bearing bone, especially femur and tibia (25). Therefore, it is meaningless to perform experiment on calvaria. In the research of Li et al., miRNA inhibitor was locally injected into mouse medullary cavity to investigate the effect of miR-188 on femoral bone formation (26). In the study of Yuan et al., PGC1β-OT1 siRNA was administrated into the mouse femur bone marrow to investigate the function of LncRNA PGC1β-OT1 on bone formation (14). Our research group also manipulated the expression of LncRNA Lnc-DIF by injecting overexpression of plasmids or siRNAs into mouse femur medullary cavity, and the regulatory effect of Lnc-DIF on bone formation was investigated by Micro-CT and Calcein labeling (27).

**Figure 1:**
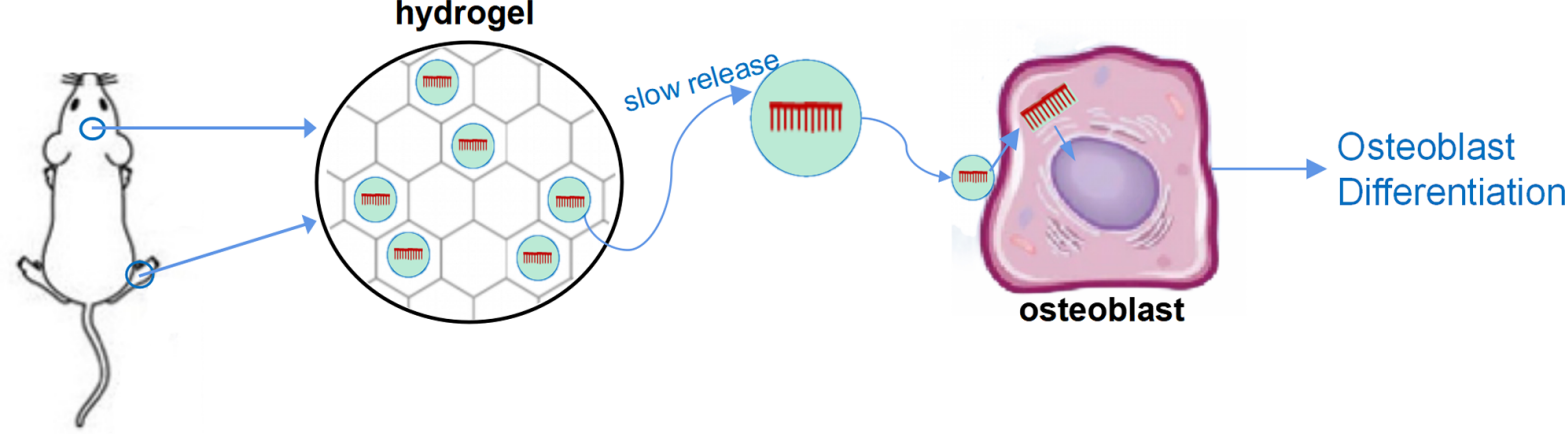
A sketch figure demonstrated the slow release effect of hydrogel. Nucleic acid was carried by vesicle-based delivery system, and the delivery system was sealed with the porous structure of the hydrogel to achieve slow release of nucleic acid drugs.

In addition, some special materials have been used for *in vivo* delivery of LncRNA to achieve sustained release on local bone formation area. In the study of Geng et al., miR-21 was encapsulated with nanocarriers and bound to the surface of titanium alloy bone implant materials, resulted in local sustained release of miR-21 while implanting titanium alloy, and, therefore, promoted osteogenesis (28, 29). Our research group utilized hyaluronic acid methacryloyl (HAMA) hydrogel to enwrap nucleic acid, which can maintain the slow-release effect of nucleic acid for 4–6 weeks *in vivo* and *in vitro*, resulting in a significant regulatory effect on bone formation (unpublished results). Moreover, both implant materials and hydrogel can combine with vesicle-based nucleic acid delivery system, including aptamer-based osteogenic targeting delivery system, exosome vesicles, and cell membrane biomimetic delivery system, and further embedded nucleic acids in the vesicles to achieve the targeted slow-release delivery of nucleic acids (30–35) (**Figure 1**).

### 4.2 Problems with LncRNA low homology

As mentioned above, LncRNA sequences have low homology between species and, therefore, could not be directly translated and applied between osteoporotic patients and animal models. To solve this problem, we have proposed several schemes.

The first approach mainly deals with extending the functions of LncRNA derived from animal models to osteoporotic patients. This solution mainly relied on extracting LncRNA functional domains and then turning them into nucleic acid medicines. For example, in our previous studies, we found that mouse-derived LncRNA AK018451 function to manipulate the level of PIEZO1 by combining with miR-103 and miR-107 (36–38). To apply this essential LncRNA on osteoporotic treatment, we synthesized the functional region of AK018451 using the novel recombinant RNA technology. Similar with AK018451 full-length sequence, the functional domain retained the function of absorbing miR-103 and miR-107. We proved that this functional sequence can perform the same function as AK018451 by transfecting it to human mesenchymal stem cell (hMSC). In the study of Li et al., HuR binding domain of LncRNA PMIF was also predicted and synthesized, and its binding effect with HuR was demonstrated. The functional region was transfected into osteoblast, and its function have been proved similar as full length of PMIF (12).This suggested that, for some functional LncRNAs, we can achieve the same function of full-length LncRNA in cells or *in vivo* by only synthesizing and modifying/transfecting functional domain sequence into cells. By this way, LncRNAs derived from other animal models can be used as a sort of RNA medicine to treat osteoporotic patients by manipulating their short functional sequences.

The second approach mainly deals with simulating functions of human-derived LncRNA on animal models to prove its function on bone formation. In another study, we identified a LncRNA fragment, named HOPE (human-derived osteogenic promoting element) that can target miR-214 (31) and performed detailed bioinformatic prediction and functional analysis. The HOPE mRNA sequence was locally transfected to bone marrow cavity of osteoporotic mice and achieved significant treatment effect. To prove the *in situ* osteogenic effect of HOPE, transfected hMSCs were locally transplanted to calvarial bone defects of mice. Results showed that bone defects with HOPE transfected hMSCs recovered significantly faster than that with non-transfected cells, indicating that HOPE can promote hMSC bone formation *in situ*. This achievement was a bold attempt for breaking through the problem of low homology of LncRNA and also brought “HOPE” to osteogentic LncRNA research. Therefore, transfecting human-derived LncRNA full-length sequence (or functional sequence, or inhibitor) into human-derived osteogenic cells and verifying its osteogenic function through *in situ* osteogenesis in model animals of bone defect might be a breakthrough to overcome *in vivo* translation experiment of human-derived LncRNA.

To optimize the experimental technology for studying the function of human LncRNA on the basis of bone defect model, our study group invented a kind of hydrogel-artificial bone material, in which HAMA was used as hydrogel to wrap porous structure composed of silk, chitosan, collagen, and hydroxyapatite. After the construction of mouse bone defect model, transfected osteogenic cells were loaded in HAMA hydrogel in the artificial bone, which enabled more accurate description of the effect of osteogenic cells on bone formation *in situ* and, thus, proved the *in situ* regulatory effect of human-derived LncRNAs on bone formation.

### 4.3 Improving potency of LncRNA

As known, miRNA regulates its target genes by binding with their 3′-Untranslated Region (UTR) sequence to inhibit translation. Therefore, it could not completely abolish the expression of target genes as traditional RNAi technology (39) or novel gene editing technology (40). In light of this, some scholars pointed out that miRNAs and related LncRNAs only can function as “decelerator” in various physiological processes. In bone formation regulation and osteoporosis treatment, their efficiency and potency were far inferior to antibody drugs (41, 42) and small-molecule compound drugs (43). Thus, improving the regulation potency of miRNAs and related LncRNAs is an important technical barrier that needs to be broken through to promote translational research studies on osteogenic LncRNAs.

Previously, most studies were focus on single-line connection between LncRNAs and miRNAs as well as between miRNAs and target genes. However, some miRNAs can regulate multiple genes on a bone formation signaling pathway, thereby exerting a stronger regulatory effect on this pathway. For example, Luo et al. discovered that miR-188 was able to regulate osteogenic and adipogenic differentiation by targeting HDAC9 and RICTOR simultaneously (26). In addition, miR-497/195 cluster was found to be able to target Fbxw7 and P4HTM to regulate bone formation via enhancing angiogenesis (44). Li et al. also reported that miR-12200 would simultaneously regulated six key genes in Wnt signaling pathway (45). We have demonstrated that miR-527 can regulate multiple core genes in several osteogenic signaling pathways and thus played regulatory roles on osteogenic differentiation and bone formation from all aspects and at various stages, launching a total war to osteogenic differentiation and bone formation.

Some LncRNAs can bind to miRNAs and block their functions and are defined as competing endogenous RNAs (ceRNAs). So far, the majority of reports about osteogenic ceRNAs have described that a LncRNA bind to a miRNA only through one binding site, which means that a LncRNA molecule can just block one miRNA molecule, which means low efficiency and specificity (46, 47).

**Figure 2:**
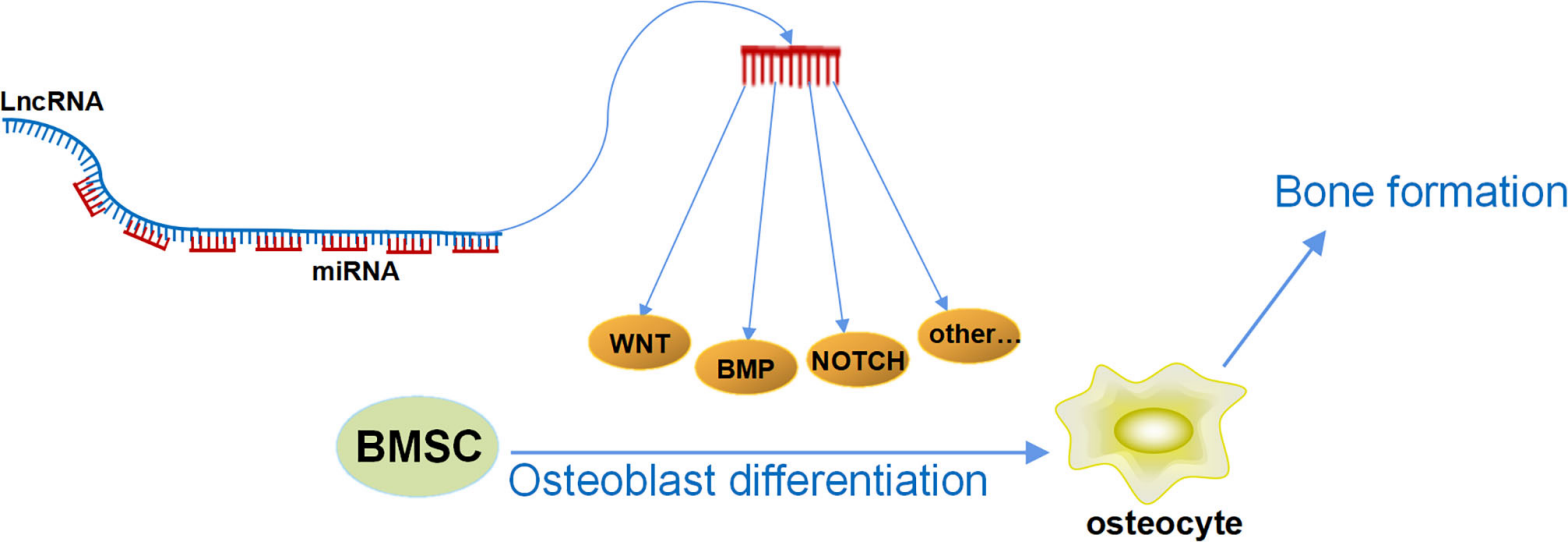
The double-amplification effect of LncRNAs. One LncRNA accurately regulates multiple miRNAs by its special repeating sequence, and each miRNA could simultaneously target multiple osteogenic mRNAs; the mRNAs would regulate multiple osteogenic signaling pathways and further regulate osteoblast differentiation and bone formation.

In our previous research, we have discovered a series of LncRNAs with special structures, which have been proven to have a strong ability in regulating osteoblast differentiation and bone formation. We identified an osteogenesis-related LncRNA that inhibited osteogenic differentiation, and, therefore, it was named Lnc-DIF (differentiation inhibiting factor). Analysis of the Lnc-DIF sequence revealed 13 sequence repeats at its end. The repeat sequence contained 53 nt, with slight differences observed in different repeat sequences. The non-repeating regions were named as “non-binding” regions, whereas repeating sequences were referred to as “binding” regions. The expression vectors containing the non-binding regions and the binding regions of Lnc-DIF were constructed, respectively, and introduced into MC3T3-E1 cells. Results showed that the Lnc-DIF binding regions containing miR-489-3p repeat binding sites can significantly inhibit osteoblast differentiation and bone formation, whereas the Lnc-DIF non-binding regions without miR-489-3p binding sites had no significant impact (17). This suggested that Lnc-DIF may be a highly efficient miRNA sponge, and one Lnc-DIF molecule may simultaneously bind and block 13 miR-489-3p, effectively inhibiting the promotion of osteoblast differentiation and bone formation by miR-489-3p. This study demonstrated that Lnc-DIF was a powerful endogenous miRNA sponge and provided an important clue that some other LncRNAs may also contain endogenous repetitive sequences that bind to miRNA. Regulating the expression level of these LncRNAs may have a significant amplification effect on their downstream miRNA levels. That is, regulating the level of one LncRNA is equivalent to regulating multiple miRNAs. Moreover, the length of the repeat sequence is short, and the selection of miRNAs is relatively more specific, thus avoiding the previous issue of traditional ceRNA that one LncRNA simultaneously regulated multiple miRNAs with different functions. This novel structure of LncRNA may become a potential precise medical strategy for the treatment of aging and postmenopausal osteoporosis in the future. At the same time, if we can find or artificially design this kind of LncRNA with repetitive sequences to regulate the multiple target miRNAs as mentioned above, then we can achieve a dual-amplification effect in that one LncRNA accurately regulates multiple miRNAs, which accurately regulate multiple bone formation–related mRNAs (**Figure 2**). This mechanism can significantly improve the potency of osteogenic LncRNA and greatly reduce its side effects, leading to efficient and accurate medical treatment in osteoporosis.

## 5 Discussion and conclusions

LncRNAs have been reported as an important regulator of osteoblast differentiation and bone formation and have increasingly attracted the attention of relevant researchers. However, because of its complex mechanisms and low homology between different species, LncRNA is rarely considered as a direct treatment for osteoporosis.

Researchers have been working on how to turn LncRNA into a useful therapy for osteoporosis. In terms of *in vivo* research, Li et al., Yuan et al., and Zhao et al. delivered miRNAs or siRNAs that target osteogenic gene into the bone marrow of mouse weight-bearing bone through local injection, resulting in enhanced bone formation (14, 26, 48). This suggested us that similar methods could be utilized to locally inject siRNA or overexpressing plasmids of LncRNA for *in vivo* experiment. Using a special CRISPR/Cas9 technique, Mo et al. managed to remove different segments of LncRNA at the genetic level to achieve gene editing of LncRNA (49, 50). This provided us with the idea that LncRNA local segments with regulatory functions could be utilized as potential nucleic acid drugs The current technology is hard to find a segment of 20–120 nt from full-length LncRNA that can bind, activate, or inactivate the target protein. However, if the target of LncRNA is miRNA, then it is easy to find the segment of LncRNA binding to miRNA and then to use the segment to fulfill its overall sequence function.

Zhao et al. investigated the regulatory function of H19 on bone formation in their research, which is homologous between human and mouse (48). However, for most LncRNA, it is difficult to find an identical LncRNA in both model animals and humans, but miRNAs with the same or similar sequence are relatively easier to find. Therefore, for LncRNA with a target gene of miRNA, as long as its downstream miRNA is homologous in humans and model animals, we can use the local functional sequence as a nucleic acid drug to achieve its regulatory effect on downstream miRNA, thereby breaking through the barrier of low homology of LncRNA. Moreover, because of the fact that some LncRNAs contain repetitive sequences, through screening for osteogenesis-related miRNAs that bind to this repetitive sequence, one can achieve molecular amplification effects that regulate multiple miRNAs by a single LncRNA. If this mRNA can target multiple osteogenic factors (14), then it can play a dual-amplification effect, making LncRNA an efficient nucleic acid drug.

We hope that the new ideas and techniques can overcome the homology issues, reducing the cost of osteogenic LncRNA research as well as exploring the advantages of osteogenic LncRNA compared with traditional drugs. Our purpose is to discover more therapeutic targets through osteogenic LncRNA research and to enhance the possibility of osteogenic LncRNA to become a therapeutic drug for osteoporosis.

LncRNAs have been reported as an important regulator of osteoblast differentiation and bone formation and have increasingly attracted the attention of relevant researchers. However, because of its complex mechanisms and low homology between different species, LncRNA is rarely considered as a direct treatment for osteoporosis.

## Author contributions

CY, FM, and AQ designed the review. CY, FM, YY, YT, and BZ wrote the manuscript. MD, KZ, and JL revised the manuscript. All authors read and approved the final manuscript.
